# Preeclampsia, Natural History, Genes, and miRNAs Associated with the Syndrome

**DOI:** 10.1155/2022/3851225

**Published:** 2022-02-14

**Authors:** Laura Parada-Niño, Luisa Fernanda Castillo-León, Adrien Morel

**Affiliations:** Center for Research in Genetics and Genomics-CIGGUR, GENIUROS Research Group, School of Medicine and Health Sciences, Universidad del Rosario, Bogotá, Colombia

## Abstract

Preeclampsia (PE) is a hypertensive disease that affects pregnant women after 20 weeks of gestation. This disease is associated with an important risk of maternal and fetal mortality. PE is described as a placental pathology because, after delivery, most women recover normal arterial pressure. Poor invasion of the spiral arteries is a phenomenon well described in PE; this leads to a hypoxic uterine bed and imbalance of antiangiogenic and proangiogenic factors in the uteroplacental region, which in turn triggers the disease phenotype. The causes of the pathology are unclear; nevertheless, numerous approaches, including next-generation sequencing, association, and case control and miRNA studies, have shed light on the genetic/molecular basis of PE. These studies help us better understand the disease to advance new treatment strategies.

## 1. Introduction

The pregnancy-specific multisystem syndrome known as preeclampsia (PE) affects 2%–10% of all gestations and is a major cause of perinatal morbidity and mortality worldwide [1, 2].

**Figure 1:**
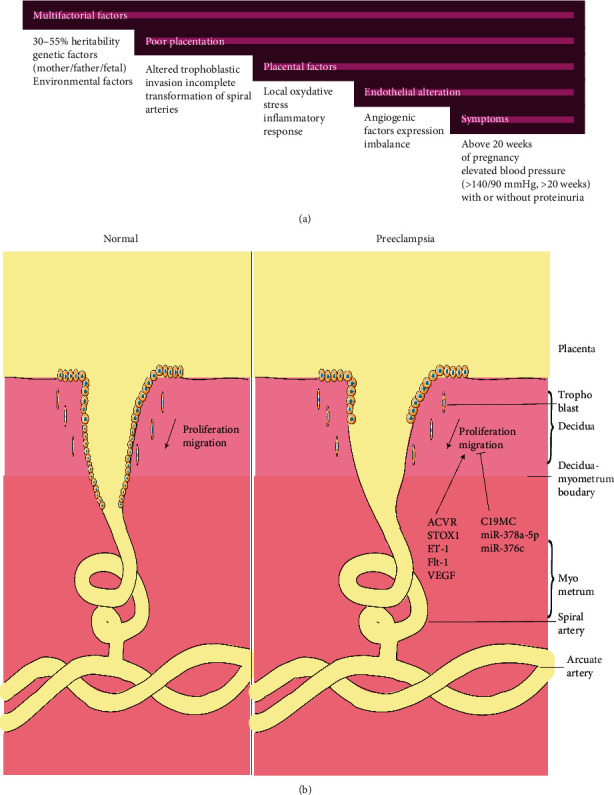
Key points of Preeclampsia (PE). A) Factors involved in PE. B) Deficient invasion of trophoblast in spiral arteries.

This disease is diagnosed above 20 weeks of gestation and is characterized usually by the presence of de novo arterial high blood pressure ≥ 140/90 mmHg (also can be superimposed with chronic hypertension), associated or not with proteinuria [3] (Figure 1(a)). Without proteinuria, the disease is diagnosed with signs of target organ damage such as serum creatinine concentration > 1.1 mg/dLor with double the baseline value in the absence of renal disease, thrombocytopenia < 100.000 plt/*μ*L, elevation of liver transaminases to twice the normal value, pulmonary edema, or symptoms of visual or cerebral disturbance [4, 5]. Women with preeclampsia have a potentially life-threatening condition, because this disease is associated with an increased risk of developing long-term complications such as cardiovascular disease [6, 7].

## 2. History of the Disease

The etiology of PE is still unclear, but studies on this topic involve the placenta in the pathophysiology, among other reasons, because it has been shown that after delivery, the signs and symptoms of the disease mostly disappear [8]. There are two subtypes of PE, an early-onset preeclampsia, also known as placental type, present in as much as 20% of PE cases and characterized by the presence of signs and symptoms before 34 weeks of gestation. This subtype has been widely linked to fetal growth restriction (FGR) [9]. Early-onset PE is characterized by abnormal trophoblastic invasion, due to incomplete transformation of the spiral arteries, leading to placental ischemia, a subsequent inflammatory response, and exacerbated oxidative stress (Figure 1(b)). All these factors contribute to a systemic imbalance of antiangiogenic and proangiogenic factors that trigger the clinical syndrome [10]. Vascular endothelial growth factor (VEGF), endoglin, and placental growth factor (PlGF) are angiogenic factors of major relevance regarding the etiology of preeclampsia [11]. On the other hand, the late-onset preeclampsia, also known as maternal subtype, develops after 34 pregnancy weeks and represents 80% of all PE cases. It has been proposed that this subtype is the consequence of a maternal genetic predisposition to cardiovascular and metabolic diseases, associated with chronic systemic inflammation combined with an altered relation between maternal blood perfusion and the increased metabolic demands from the placenta and the fetus. These disturbances can lead to placental dysfunction and associated placental hypoperfusion, without poor placentation, as seen in early-onset preeclampsia [9, 12].

Currently, prophylactic treatment with low-dose aspirin (<100 mg/day) is the conventional therapy for preventing the onset of signs and symptoms, as well as complications related to preeclampsia. Prophylactic treatment beginning before 16 weeks of gestation can reduce PE incidence and rate of complications by up to 70% [13].

## 3. Association Studies

Preeclampsia (PE) is a multifactorial pregnancy-specific syndrome without a Mendelian inheritance pattern [14–17]. It is the consequence of complex interactions between two or more maternal and fetal genes, in addition to the environment [18]. Several pedigree analyses in different populations reveal clear heritability, which is estimated between 31% and 54% [19–21]. 35% of the variation in PE can be attributed to maternal genetics, 20% to fetal genetics, 13% to paternal genetics, and 32% to environmental factors [22].

Currently, the etiology of PE is not clear; therefore, several association studies have been developed, including the study of candidate genes, genome-wide association studies (GWAS), and linkage to identify susceptibility genes to PE [14, 23–26]. However, a limitation of these studies is that they are mainly focused on analyzing the mother, even though it is a disease involving genetic factors of both parents.

Candidate gene studies have compared the frequency of genetic variations between cases and controls to determine genetic associations. Most studies analyze a single polymorphism in a candidate gene, while a minority analyzes several genes or multiple polymorphisms in one or more genes. These genes have been selected based on the current understanding of PE pathology [11, 24], such as genes involved in endothelial functions and related with blood pressure regulation and key genes involved in the regulation of blood pressure: *sVEFGR-1*, *TGF-β*, *Eng*, *RAS*, *AGT*, *ACE*, *AGTR1* [27, 28], and *eNOS* [29, 30]; genes that regulate lipid metabolism and oxidative stress: *EPHX1* [31, 32], *GST*, *NOX1*, *SOD2* [18, 33], *APOE* [27], *LPL* [34], and *ROS* [35, 36]; and thrombophilic genes involved in coagulation: *F5*, *F2*, and *MTHFR* [27, 28, 37–39]. As previously described, PE can be caused by genetic factors from both parents, an observation supported by Andraweera et al. and Zusterzeel et al., who found that variants present in the father, more precisely in the *VEGF*, *PIGF*, and *GST1* genes, can double the risk of PE [40, 41]. Studies suggest that PE is an immune maladaptation due to the defective interaction between fetal antigens and maternal immune cells, limiting the establishment of immune tolerance to normal placentation. The genes *TNFα* [42, 43], *IFN-γ*, *IL-1*, *IL-4*, *IL-10* [44–46], IL-*27* [47, 48], *HLA-G*, *TGF-β*, *E*, *HLA-C2*, and *KIR* are involved in this immune response (Table 1).

Genetic mapping and linkage studies conducted in family pedigrees have identified chromosomal regions associated with PE in all chromosomes, with chromosome 2 having the highest number of *loci* involved. For example, *ACVR2* and *STOX1*, the two most studied candidate genes, were identified using genetic linkage methods. The first was identified in a case-control study in which Moses and collaborators found a >10-fold differential gene expression in *decidua* samples between PE and normotensive women [49]. For the second one, the analysis of 67 affected families showed an association between *STOX1* and the disease [50].

Regarding GWAS, an approach designed to study case and control cohorts of unrelated individuals, multiple *loci* that can explain a portion of the variation in preeclampsia phenotypes have been identified (Table 2). For example, the inhibin beta B gene (*INHBB*), a pituitary follicle-stimulating hormone secretion inhibitor that has been suggested to play a role in PE development [51], and the pregnancy-specific beta-1 glycoprotein 11 (*PSG11*) [49], which is mainly produced by placental syncytiotrophoblast during pregnancy, showed a high association. The *MTHFR* gene was associated with a protective effect against PE in an association study where 816 nonpreeclamptic women were significantly less prone to develop preeclampsia than 1006 preeclamptic women [52]; with regard to an enzyme involved in the regulation of folate metabolism, McGinnis and collaborators performed the first GWAS study in a cohort of descendants composed of 4380 cases (631 early and 3749 late-onset PE) and 310,238 controls. They discovered a sequence variant in the fetal genome that may increase the risk for PE near to the *sFlt1* gene, an antiangiogenic factor [53, 54]. In this gene, the variant rs4769613 is a preeclampsia-specific risk factor, when present in the placenta genome but not directly in the parental genotype [53, 55]. However, the placental genotype for rs4769613 combined with clinical parameters may contribute to early identification of high-risk women and may provide insight into how altered expression relates to the pathophysiology of preeclampsia and its subtypes.

Another way to identify genes involved in the pathogenesis of a multifactorial disease such as PE is through microarray analysis to study the expression patterns of tens of thousands of genes simultaneously [14]. To date, these studies have been performed with RNA taken from placental biopsies in the second and third trimesters, placenta samples up to one hour after delivery, decidua tissue, and cytotrophoblast cultures (Table 3). These studies revealed that genes related to obesity, cytokine-receptor genes, and genes related to apoptosis could be potential susceptibility genes, based on the altered expression in preeclamptic compared with normotensive women [56, 57]. Previous studies have shown that genes involved in activin/inhibin signalling (*ACVR1*, *ACVR1C*, *ACVR2A*, *INHA*, *INHBB*), structural components (*COL4A1*, *COL4A2*), and aminopeptidases (*ERAP1*, *ERAP2*, and *LNPEP*) were differentially expressed in the maternal-fetal interface of PE pregnant women [23, 58].

Despite the large number of candidate genes and associated *loci* identified as potential candidates, a universal susceptibility gene has not been found, principally due to the lack of reproducibility [14]. For example, there is substantial evidence suggesting the role of *STOX1* as a potential PE susceptibility gene; linkage studies allowed a detailed analysis of this gene and several mutations were found and tested in *in vitro* and *in vivo* experiments, confirming that one of these variants could be a risk factor for PE [50]. Nevertheless, Iglesias-Platas et al. could not replicate these results [59]. Therefore, the experimental design should be standardized considering the case definition, sample size, and statistical significance. Combining genome-wide studies with the candidate gene approaches in other population samples, subsequently validating the findings would increase the statistical power to identify potential genes useful for diagnosis. Given the genetic heterogeneity of the disease and the variation between different ethnic populations, it is difficult to identify a unique gene.

## 4. miRNAs

miRNAs are small noncoding RNA of 18-22 nucleotides which are involved in post-transcriptional regulation. Since its discovery in 1993, miRNAs have been described in numerous biological processes, such as proliferation, cell growth, and embryogenesis [60, 61]. miRNAs can induce mRNA destabilization and/or inhibition of translation. miRNAs play an important role in cellular life, as it has been demonstrated that the loss of miRNA biogenesis is lethal during embryonic development [62, 63]. The molecular mechanisms have been well described. RNA pol II is involved in the synthesis of miRNAs in *pre*-miRNA. The latter undergoes a maturation process by Drosha/DGCR8 in *pre*-miRNA, exported from the nucleus into the cytoplasm through exportin-5. This pre-miRNA is processed by the DICER enzyme into the miRNA duplex, and finally, one of the two strands is incorporated into a complex, the RNA-induced silencing complex (RISC). This complex can bind to a specific 3′UTR to play its role in regulating mRNA posttranscription [64].

Given the principal role of miRNAs in controlling mRNA expression, it is not surprising that the dysregulation of their expression leads to the loss of cellular homeostasis and consequently to disease emergence. Different studies have revealed the involvement of miRNAs in the regulation of placental function and development. The evolution of miRNA expression analysis techniques has helped to describe and/or understand miRNA function in healthy placentas. Dysregulation of miRNA expression has been reported in several diseases and more specifically in PE [65–69]. There are more than 2000 miRNAs in the human genome, and it is estimated that over 382 miRNAs are expressed in trophoblasts isolated from normal human placenta [64, 70].

PE, as described previously, is characterized by hypertension occurring after the twentieth week of pregnancy, and it is an important cause of maternal and child morbidity and mortality. Different molecular hypotheses have been proposed to explain the origin of this pathology, and recently, some miRNAs have been demonstrated to be involved in the syndrome due to the function of several miRNA clusters in the placenta.

C19MC, the largest cluster described, is composed of 46 miRNA genes that represent 59 mature miRNAs [71, 72]. C19MC miRNAs are highly expressed in the placenta, more precisely in trophoblasts, and in preeclampsia, a decrease in C19MC miRNA expression has been reported [73, 74]. It has been described that C19MC miRNA expression is increased from the first to third trimester of pregnancy under normal conditions [70]. Hromadnikova et al. in 2017 showed an increase in C19MC miRNA expression, more specifically miR-517-5p, miR-518b, and miR-520h in women with PE versus women with normal pregnancy [75]. Recently, Zhang et al. showed that a member of C19MC, miR-515-5p, targets and inhibits the expression of *CYP19A1* and *GCM1*, among other genes important in trophoblast differentiation [76]. Besides, Xie et al. showed that C19MC miRNAs play an inhibitory role in trophoblast migration [73]. Probably, the increase of the last ones is dysregulated and can induce a decontrolled inhibition of trophoblasts migration. Furthermore, the generation of C19MC double homozygous transgenic mice lead to C19MC miRNA overexpression [132] causing a change of expression of mRNAs involved in cell migration as *Mmp1a* or *Prl5a1*. These expression changes probably conduce to malformation of the placenta spotted by an irregular boundary between the labyrinthine layer and the junctional zone. The authors confirmed these observations by using probes for *Tpbpb* and *Ceacam11* (more expressed in spongiotrophoblast cells) invagination of spongiotrophoblast cells in the labyrinth layer [73], which supports the important role of C19MC miRNAs in cell migration regulation. Interestingly, C19MC is expressed from the paternal chromosome, while C14MC, another large miRNA cluster located on the chromosome 14q32 locus, is expressed from the maternal chromosome [77, 78]. The last one contains 52 miRNAs, and different studies have shown the importance of C14MC in placentation. In a mouse model, the KO of miR-127 affects the regulation of *Rtl1*, which disturbs placental development [79]. Furthermore, trophoblast proliferation, invasion, and migration are regulated by miR-378a-5p and miR-376c, among others. The expression of these miRNAs was downregulated in women with PE. miR-378a-5p directly targets and represses the expression of NODAL proteins [80]. miR-376c is involved in the inhibition of NODAL signalling by targeting ALK5 and ALK7, proteins that are important in this pathway [81]. These results suggest that dysregulation of NODAL by miRNAs can prevent trophoblast migration, invasion, and proliferation in women with PE.

Furthermore, other studies have shown a disturbance of miRNA expression in PE, and their functions are well documented. Among these, compromise of processes such as cell cycle, migration, and angiogenesis can explain the PE phenotype [82]. Recently, some researchers have found variants in the 3′UTR of *PTX3*, *RGS2*, and *MTHFR* genes, which are associated with PE [83–85]. The involvement of these proteins in PE has been previously described. The decrease in RGS2 expression contributes to PE through its inhibitory function on arterial pressure regulation [89], and PTX3 is involved in the regulation of inflammation and is upregulated in women with PE [86]. Variants in MTHFR can induce a decrease in the enzymatic activity that increases folate levels, which contributes to PE [87]. Variants in the 3′UTR may cause dysregulation of gene expression through loss or gain of miRNA recognition, as has been described for other diseases such as epilepsy [88] and venous thrombosis [133], and as a susceptibility factor in cancer [90–92]. PE is a multifactorial disease for which the genetic load induces the PE phenotype, and the analysis of noncoding region as 3′UTR can help to better understand the syndrome.

## 5. Unconventional Therapeutic Strategies in Preeclampsia

In addition to traditional medical management for preeclampsia, other forms of treatment have been proposed and open a future with new perspectives of control for the signs and symptoms of this disease, as well as for the prevention of maternal and fetal comorbidities triggered by preeclampsia.

### 5.1. Mitochondrial Antioxidant Therapy

Excessive production of reactive oxygen species (ROS) has been linked to pathological processes such as preeclampsia, secondary to the altered placentation that occurs in this disease, which triggers an increased inflammatory response and endothelial dysfunction. Different sources of ROS have been identified in the placenta, among which mitochondrial dysfunction may be related, and this alteration has been linked to the increased production of these compounds in the pathophysiology of preeclampsia [93].

Mitochondria-targeted antioxidants have been proposed as a treatment to reverse the effects of mitochondrial oxidative stress in the placenta. The use of the mitochondrial-specific hydrogen sulfide donor AP39 in cellular models has been shown to prevent ROS production and reverse mitochondrial oxidative stress, thereby decreasing the activation of hypoxia-inducible transcription factors such as HIF-1*α*, as well as enhancing cytochrome C oxidase levels and reversing the antiangiogenic response in hypoxic trophoblast cells. However, further studies are required to demonstrate whether the effects of AP39 can improve symptomatology in animal models of preeclampsia [94].

### 5.2. Ligands of sFLT1 as a Therapeutic Target

Overexpression of the antiangiogenic factor sFLT1 (vascular endothelial growth factor receptor 1) has been linked to production of endothelial damage characteristic of preeclampsia, proteinuria, hypertension, and target organ damage. Several studies have identified sFLT1 as a therapeutic target for the management of preeclampsia [95]. In primate animal models, for example, the recombinant sFLT1 ligand placental growth factor (PlGF) has been shown to reduce blood pressure and proteinuria levels compared to untreated preeclamptic controls. In the case of VEGF121, another recombinant ligand of sFLT1, this factor showed improvement in preeclampsia-related symptomatology in animal models [96]. Therefore, the use of these strategies offers an alternative for the management and control of the symptomatology attributed to PE, which could lead to an improvement in the associated morbidity and mortality.

### 5.3. RNAi in Animal Models

Preeclampsia pathogenesis has been associated with dysfunction of the renin-angiotensin-aldosterone (R-A-A) axis, resulting in decreased renin levels, increased angiotensin-converting enzyme (ACE) activity, decreased angiotensin II levels, and increased response to this enzyme. This axis has been implicated in the regulation of blood pressure and water-electrolyte balance, and its components are expressed among other tissues, in spiral arteries, human placenta, and fetal tissues; therefore, alterations in this axis represent a risk factor for the development of PE [97]. Some mutations on the R-A-A axis have been associated with preeclampsia, as in the case of the missense mutation in the *AGT* (angiotensin) gene M235T, which was associated with elevated plasma levels of AGT in carriers of this variant [98].

The use of RNAi directed against maternal hepatic TGA offers an alternative for the symptomatic control of preeclampsia in two animal models tested by German investigators. In one of these models, female mice expressing human Agt were crossed with male mice expressing human renin, which resulted in the upregulation of the R-A-A axis at central circulatory and uteroplacental levels. In the second model, the researchers surgically decreased uterine perfusion pressure and thus induced placental ischemia, which secondarily produced alteration of the R-A-A axis with hypertension, oxidative stress, excess of inflammatory factors at the circulatory level, and decreased glomerular function. They found that the use of siRNA in the two animal models produced an improvement in the signs and symptoms characteristic of preeclampsia, with reduction of blood pressure, improvement of renal function, and intrauterine growth restriction (IUGR) patterns. [99]. With further studies, this result might open an opportunity to add new tools to avoid complications caused by preeclampsia.

### 5.4. PP13 as a Future Tool in Preeclampsia Management

Galectins are a family of carbohydrate binding proteins with affinity to *β*-galactoside sugars, and one of them, Galectin 13, also known as protein 13 (PP13), is expressed by the syncytiotrophoblast and detected since early stages of pregnancy. This protein has been associated with critical processes of pregnancy such as placental implantation and vascular transformation [100]. In a functional *in vivo* study using pregnant rats, the authors demonstrated that intravenous injections of PP13 could reduce blood pressure [101]. Also, a subsequent study with implantation of osmotic pumps in nongravid rats injected subcutaneously with recombinant PP13 (rPP13) showed that uterine vessels expanded significantly, demonstrating that PP13 could have an important role as a future tool in the treatment of PE [102, 103].

## 6. Conclusion

Preeclampsia is a complex disease constituting a serious public health problem. Numerous published studies on the subject have described different genes involved in the molecular etiopathology of this disease; nevertheless, to date, no universal genes have been described. Furthermore, since the last decade, miRNAs have been described to be involved in the molecular mechanism of the disease. The complexity of multifactorial diseases makes it difficult to resolve and find a treatment, and there is still much to be discovered about the pathophysiology and treatment of this illness. Importantly, the majority of studies have been focused on the coding region of candidate genes, neglecting noncoding sequences, such as promoters and/or untranslated regions. These sequences might be critical to understanding the role of genetic factors in PE.

## Figures and Tables

**Table 1:** Genes and SNPs associated with preeclampsia.

Gene	SNP	Study size (case/control)	Sample origin	Association	Reference
*IL1RL1*	rs1420103	214/208	Mother	GG increases mild preeclampsia risk; TT reduces late-onset severe preeclampsia	[47]
	rs13017455			CT reduces preeclampsia risk	
*ACVR2A*	rs1424954	613/693	Mother	These SNPs were associated with the more severe early onset preeclampsia	[104]
*MTHFR*	rs1801131	150/150	Mother	CC genotype increases risk	[105]
		192/196	Mother	The synergic (rs1801131+rs1801133) effect of MTHFR variants could increase PE and EOPE risk	[106]
	rs1801133				
		198/101	Mother	Increased risk	[107]
		125/274	Mother	Protective effect	[108]
*IL-1B*	rs1143630	169/287	Mother	“T” allele increase risk	[109]
*C6*	rs7444800	203/233	Mother	Genetic variations in this gene increase risk, and the risk varied by preeclampsia subtypes	[110]
	rs4957381				
*MASP1*	rs1108450				
	rs3774282				
	rs698106				
*VEGF* and *VEGFR1*	*VEGFR1* rs722503	165/191	Mother	Increase risk after the age of 40 years	[111]
	*VEGFA* rs3025039			C allele increase risk; T allele protective allele	
*HLA-G*	rs17179101 and rs9380142	47/68	Mother	This SNP combination increases risk	[112]
	HLA-G 0106G	83/240	Mother	Increased risk	[113]
*IL-10*	rs1800896	151/189	Mother	Increased risk	[44]
		134/164	Mother	Increased risk	[45]
		101/91	Mother	AA allele increases risk	[46]
*ICOS*	rs4675378	130/260	Mother	T allele and TT genotype protective allele	[114]
*GSTP1*	rs1695	113 trios/317 (149 men, 168 women)	Mother/father/child	This polymorphism in paternal and fetal GSTP1 gene increases risk	[41]
		125/274	Mother	G allele protective effect	[108]
*TGF-B1*	rs1800470	175/253	Mother	TT genotype protective allele	[115]
*IL-6*	rs1800795	116/107	Mother	C allele protective allele	[116]
*NLRP1*	rs12150220	286/309	Mother	Risk effect	[117]
*eNOS*	rs1799983	322/522	Mother	Risk effect	[118]
	rs2297518	353/212	Mother	A allele increases risk	[119]
*IL-1A*	rs3783550	79/210	Mother	A allele increases risk	[120]
*ACE*	rs1799752	66/37	Mother	DD genotype and D allele increase risk	[121]
*EDN1*	rs5370	61/49	Mother/father	rs5370 SNP protective effect	[122]
*ERAP2*	rs2549782	528/575	Mother and neonates	Risk effect	[123]
*GSTM1*	rs4025935	Mother	112/233	*GSTT1* deletion and combined *GSTM1/GSTT1* deletion increase risk	[124]
*GSTT1*	rs71748309	Mother	112/233		

**Table 2:** Genes associated with preeclampsia by GWAS.

Gene	SNP	Sample origin	Study size (case/control)	Effect	Reference
*MTHFR*	rs17367504	Mother	1006/816	Protective effect	[52]
*ATP2B1*	rs17249754a	Mother	1006/816	Protective effect	
*PAX5*	rs16933812a	Mother	1006/816	Protective effect	
*PLCD3*	rs12946454a	Mother	1006/816	Protective effect	
*INHIBIN*	rs7579169	Mother	538/540	Risk effect	[51]
*KIAA1239*	rs1426409	Mother	177/116	Risk effect	[125]
*ESRRG*	rs17686866	Mother	177/116	Risk effect	
*LMCD1*	rs9831647	Mother	177/116	Risk effect	
*IFLTD1*	rs10743565	Mother	177/116	Risk effect	
*PSG11*	rs10412348	Mother	177/116	Risk effect	
*FGF14*	rs11617740	Mother	137/2986	Multicenter study: none detected SNP reached Bonferroni-corrected significance due to the small number of cases	[126]
*C21orf121*	rs2839440	Mother	137/2986		
*MGC45800*	rs12641856	Mother	137/2986		
*MCM8*	rs4815879	Mother	137/2986		
*MUC21*	rs28360974	Mother	137/2986		
*BAMBI*	rs1248993	Mother	137/2986		
*NPVF*	rs975369	Mother	137/2986		
*ADRA1D*	rs1556832	Mother	137/2986		
*SCN2B*	rs11600901	Mother	137/2986		
*MYCBP2*	rs7322722	Mother	137/2986		
*INVS*	rs10989019	Mother	137/2986		
*FLT1*	rs4769613	Child	4380/310,238	C allele increases risk effect	[53]
*PLEKHG1*	rs9478812	Mother	877/2004	Risk effect	[127]

**Table 3:** Genes associated with PE by microarrays studies.

Gene	Study size	Tissue	Fold change	Reference
*PRG2*	4/4	Syncytiotrophoblast severe preeclampsia (placenta)	6.4	[128]
*FSTL3*	4/4		3.7	
*FABP4*	4/4		3.5	
*RDH13*	4/4		3.1	
*PLAC1*	4/4		-3.4	
*SERPINI1*	4/4		-4.4	
*GSTA3*	4/4		-4.8	

*CXCL8*	3/4	Cytotrophoblast severe preeclampsia (placenta)	3.5	[129]
*GH2*	3/4		-14.2	

*sFLT1*	21/21	Placenta (full thickness)	2.6	[58]
*ENG*	23/37		1.852.919.237	
*INHIBIN*	21/21		3.0	[58, 130, 131]
*SIAE*	23/37		1.354.912.795	[58]
*PAPPA2*	21/21		2.9	
*BRSK2*	21/21		8.6	
*FLJ90650*	21/21		10.0	
*LEP*	21/21		40.0	
*LHB*	21/21		4.1	
*PDGFD*	21/21		-2.3	
*COX17*	21/21		-4.3	

*HS3ST2*	60/65	Maternal placenta (decidua basalis)	-1.39	[130]
*TNFRSF14*	60/65		-1.35	
*SLC2A6*	60/65		-1.32	
*DPP7*	60/65		-1.22	
*CD72*	60/65		-1.21	
*PER3*	60/65		-1.20	
*DBP*	60/65		-1.20	
*PDK4*	60/65		1.72	
*HS3ST2*	60/65		-1.39	

*COL17A1*	6/6	Placental biopsies	6.6	[56]
*KRT2*	6/6		6.4	
*NCAM1*	6/6		3.8	
*PKP2*	6/6		3.2	
*BMP2*	6/6		-3.9	
*CDH3*	6/6		-4.3	
*LEP OB/OBS*	6/6		43.6	
*MEF2A*	6/6		3.9	
*MUC1*	6/6		3.6	
